# Somatostatin receptor imaging by SPECT and PET in patients with chronic inflammatory disorders: a systematic review

**DOI:** 10.1007/s00259-019-04489-z

**Published:** 2019-08-28

**Authors:** Luz Kelly Anzola, Andor W. J. M. Glaudemans, Rudi A. J. O. Dierckx, F. Andres Martinez, Sergio Moreno, Alberto Signore

**Affiliations:** 1 Nuclear Medicine Unit, Clinica Reina Sofia, Bogotà, Colombia; 2 Medical Imaging Center, Department of Nuclear Medicine and Molecular Imaging, University of Groningen, University Medical Center Groningen, Groningen, The Netherlands; 3 Radiology Department Clinica Reina Sofia, Bogotà, Colombia; 4 Clinical Epidemiologist Universidad Nacional de Colombia, Bogota, Colombia; 5 Nuclear Medicine Unit, Department of Medical-Surgical Sciences and of Translational Medicine, Faculty of Medicine and Psychology, “Sapienza” University, Rome, Italy

**Keywords:** Somatostatin receptor imaging, SPECT, PET, Inflammatory diseases

## Abstract

**Objective:**

To review the literature on the clinical application of radiolabeled somatostatin receptor scintigraphy (SRS) by SPECT and PET in adults with chronic inflammatory diseases.

**Research design:**

Systematic review of published observational studies between 1993 and 2017.

**Data collection and analysis:**

The Cochrane Central Register of Controlled Trials, MedLine, EMBASE, PubMed, Google Scholar, OVID, EBSCO, Scopus, and Web of Science were used to search for studies on the use of SRS in adults with chronic inflammatory diseases. A team of reviewers independently screened for eligible studies. Quality of evidence was assessed by QUADAS approach.

**Results:**

Eligible papers included 38 studies. Studied populations were heterogeneous, and patients were classified according to the diagnosed disease: endothelial inflammation, rheumatoid arthritis, cardiac allograft rejection, granulomatous diseases, small vessel vasculitis, idiopathic pulmonary fibrosis, sarcoidosis, and thyroid exophthalmopathy. Because of many quality differences between studies, it was not possible to pool data, and a narrative synthesis is reported.

**Conclusion:**

Results highlight the value of SRS to detect active inflammation in several chronic inflammatory conditions, despite the bias related to the index test, showing lack of standardization of the scintigraphic technique and high variability of methods used to clinically evaluate inflammatory condition.

## Introduction

Chronic inflammatory diseases are characterized by long-standing mononuclear cell infiltration of the target organ, leading to hypofunction and requiring life-long treatment [1]. In the past years, several different radiopharmaceuticals have been synthetized for molecular nuclear medicine that may contribute to the diagnosis and prognosis of these diseases [2]. In particular, several peptides, receptor ligands, and monoclonal antibodies have been radiolabeled, allowing the in vivo visualization of the inflammatory process at cellular and molecular level [3].

As far as radiolabeled peptides are concerned, it is important to emphasize the increasing use of somatostatin (SST) analogues targeting somatostatin receptors (SSTR) in inflammatory diseases, particularly in rheumatoid arthritis (RA), Sjögren syndrome (SS), and autoimmune thyroid diseases. Because the broader spectrum of interaction with SSTR in other different pathological conditions than neuroendocrine tumors, such as chronic inflammation, and because the known presence of SSTR over-expression by inflammatory cells, immunological cells in different tissues, blood vessels among others, it has been possible to use different radiolabeled somatostatin analogues with diverse affinity for these receptors, for diagnostic purposes in different oncological and inflammatory scenarios [4]. Different SST analogues have been proposed in clinical practice. Among all synthetic compounds pentetreotide (Octreotide®), labeled with ^111^In, was the most widely used for imaging purposes, because of its high affinity for SSTR 2 and 5. New radiopharmaceuticals for somatostatin receptor scintigraphy (SRS), showing a different and/or wider affinity to the receptors, are now available and labeling has been obtained both with gamma and positron emitters for diagnostic purposes, with SPECT or PET, respectively, such as ^68^Ga-DOTA-TATE and ^64^Cu-DOTA-TATE(affinity to type 2 receptors), ^68^Ga-DOTA-TOC (more selective to 2 and 5 type receptors), ^68^Ga-DOTA-NOC (affinity to 2,3 and 5 type receptors), and 99mTc-EDDA/HYNIC-TOC (affinity to 2 and 5 type receptors) [5].

The labeling of octreotide and other analogues has been carried out by conjugation of the peptide with a bi-functional chelator, DTPA, DOTA, or NOTA [6], and subsequent addition of a radionuclide such as ^123^I, ^111^In (pentreotide), ^99m^Tc (depreotide-EDDA/HYNIC-TOC) or ^68^Ga, ^18^F, and ^64^Cu (DOTATATE) [7].

SST was first isolated from ovine hypothalamic extracts and was characterized as a tetradecapetide. It was identified as part of the releasing hormone family for its property to inhibit the secretion of growth hormone from pituitary cells by Brazeau and colleagues in 1973 [8]. SST-producing cells are typically neurons or endocrine-like cells and are found in high density in the central and peripheral nervous systems and in the endocrine pancreas and in the gut and in small numbers in the thyroid, adrenals, submandibular glands, kidneys, prostate, placenta blood vessel walls, and immune cells [9]. It is a cyclic hormone that regulates several physiological cell processes via specific receptors (SSTR) which are expressed by nerve cells, many neuroendocrine cells and inflammatory cells such as lymphocytes, monocytes, monocytes/macrophages, peripheral blood mononuclear cells and thymocytes [10]. Five SSTR subtypes have been cloned which have been found in cells involved in inflammatory responses with high density expression observed on neoangiogenic and peritumoral vessels, epithelioid cells, proliferating synovial vessels, and activated lymphocytes and monocytes [11]. In the peripheral blood mononuclear cells and in the spleen, mainly SSTR subtypes 2 and 3 are found; in the thymus, mainly SSTR subtypes 1, 2, and 3; in the macrophages and in dendritic cells, mainly SSTR subtype 2; in B lymphocytes, mainly SSTR subtype 3; and in T lymphocytes, SSTR subtypes 1 to 5 [4]. For diagnostic purposes, radiolabeled SST analogues have been used in SPECT and PET with clinically relevant results in different chronic inflammatory diseases, particularly for the evaluation of disease activity, prognosis, and therapy follow-up [12, 13]. Although 18F-fluorodeoxyglucose (18F-FDG) PET/CT has recently gained a role also in infective and inflammatory disease, because logistically it is easier and quicker to perform, this radiopharmaceutical is not specific and therefore it is not able to discriminate an infection from an inflammation [14].

In this review, we systematically analyze all papers on SRS (with either SPECT or PET) in the setting of various chronic inflammatory diseases: endothelial inflammation, rheumatoid arthritis, cardiac allograft rejection, small vessel vasculitis, idiopathic pulmonary fibrosis, sarcoidosis and granulomatous diseases, and Graves’ ophthalmopathy.

### Why it is important to do this review

It important to do this review because the literature has shown the potential utility of radiolabeled somatostatin analogues not only in the diagnostic setting but as prognostic factor and as part of treatment control in some chronic inflammatory diseases, it is important to assess and collect the best evidence published up today regarding to this topic.

## Methods

### Criteria for considering studies for this review

#### Types of studies

Observational cohort studies, systematic reviews, reports of more than three cases.

#### Types of participants

Adult patients with any diagnosed inflammatory chronic disease such as rheumatoid arthritis, sarcoidosis, cardiac allograft, atherosclerotic plaques, Sjögren disease, idiopathic pulmonary fibrosis, Graves’ exophthalmopathy who had a radiolabeled somatostatin receptor analogue scintigraphy for diagnostic purpose.

#### Types of intervention

Somatostatin receptor analogues radiolabeled with Spect tracers: ^111^In octreotide, ^99m^Tc:^99^Tc-EDDA/HYNIC-TOC, ^99m^Tc P829 and with PET tracers: ^68^GaDOTA-NOC, ^68^Ga DOTA-TOC, ^68^Ga DOTA-TATE, and 64CuDOTATATE.

#### Types of study outcome

The final diagnostic for the entire population was related to chronic inflammatory diseases such as: rheumatoid arthritis, Sjögren disease, Graves’ exophthalmopathy, atherosclerotic plaques, idiopathic pulmonary fibrosis, sarcoidosis, and other inflammatory conditions.

### Search method for identifying the eligible studies

The selection of appropriate publications was based on the PRISMA guidelines [15]. We searched in Cochrane Central Register of Controlled Trials (CENTRAL) published in the Cochrane Library, MedLine (1993–2017), EMBASE (1993–2017), PubMed (1993–2017), Google Scholar (1993–2017), OVID (1993–2017), EBSCO (1993–2017), and Scopus and Web of Science (1993–2017). All relevant studies published in English, Italian, and Spanish language were identified by using the following search strategy: (“receptors, somatostatin”[MeSH Terms] OR “receptors”[All Fields] AND “somatostatin”[All Fields]) OR (“somatostatin receptors”[All Fields] OR (“somatostatin”[All Fields] AND “receptor”[All Fields]) OR “somatostatin receptor”[All Fields]) AND (“Bildgebung”[Journal] OR “imaging”[All Fields]) AND (“adult”[MeSH Terms] OR “adult”[All Fields] OR “adults”[All Fields]) NOT (“neoplasms”[MeSH Terms] OR “neoplasms”[All Fields] OR “neoplasm”[All Fields]) NOT (“tumors”[All Fields] OR “neoplasms”[MeSH Terms] OR “neoplasms”[All Fields] OR “tumors”[All Fields]) somatostatin receptor imaging AND adults.

### Inclusion criteria

Studies or reports in which compounds were labeled with ^68^Ga, ^99m^Tc, ^68^Ga, and ^64^Cu for applications other than in oncology-related indications were included. Every effort was made to include both the earliest and the most recent publications relating to a particular application, as well as any study with a significant new contribution. The decision to include or exclude an article was made by consensus.

### Exclusion criteria

Publications in which the focus was on SRS in oncology-related applications or in which the emphasis was on aspects related to generators, radiochemistry, animal models, experimental reports, or physics were excluded.

Literature search was broadened to all reference lists of all retrieved articles, including observational studies, systematic reviews, conference proceedings, and poster presentations. Studies or reports with more than three cases in which SRS was used in patients with inflammatory diseases were also included. Case reports allusive to just one patient where not taken in consideration because the results of the observations could not be compared between patients in order to highlight differences or similarities.

### Selection of studies data extraction and management

The decision to include or exclude an article was made by consensus between LKF, AWJMG, AS, and AM. We used the search strategies described to obtain titles and abstracts of studies of potential relevance to the review.

Two authors, one with expertise in the topic (LKAF), the second with expertise in the statistical methodology (SM), undertook the search. LKF, SM, and AM screened independently titles, abstracts, conferences proceedings, posters, and full texts for eligibility. We ensured the avoidance of multiple studies reporting on the same patient population by controlling the duplicate records in a peer review way, including the entire databases results; we also used standardized, pilot-tested forms, together with detailed instructions. Reviewers resolved disagreement through discussion or, if required, by adjudication by a third reviewer (SM).

### Assessment of risk bias in included studies

The included studies were assessed by using the Cochrane Collaboration’s tool [16] and Quadas-2 [17] for potential source of bias and variation: patient selection bias (study design, patient recruitment, prospective data collection, consecutive patient enrollment), patient variation (demographic features, disease prevalence, disease severity, prior testing), index test bias (test review bias, threshold selection), index test variation (observer variation, availability of clinical information, test technology, test execution), reference standard bias (inappropriate reference standard, diagnostic review bias, incorporation bias), reference standard variation (definition of target condition), flow and timing bias (disease progression bias, treatment paradox, partial verification bias, differential verification bias, withdrawals, uninterpretable test results, sample size).

### Data synthesis

Because of differences between studies, it was not possible to pool data, and so a narrative synthesis was prepared. Study population was very heterogeneous, for this reason we classified patients according to the diagnosed disease: endothelial inflammation and atherosclerosis, rheumatoid arthritis, idiopathic pulmonary fibrosis, Graves’ ophthalmopathy, sarcoidosis, and other chronic inflammatory diseases.

**Fig. 1:**
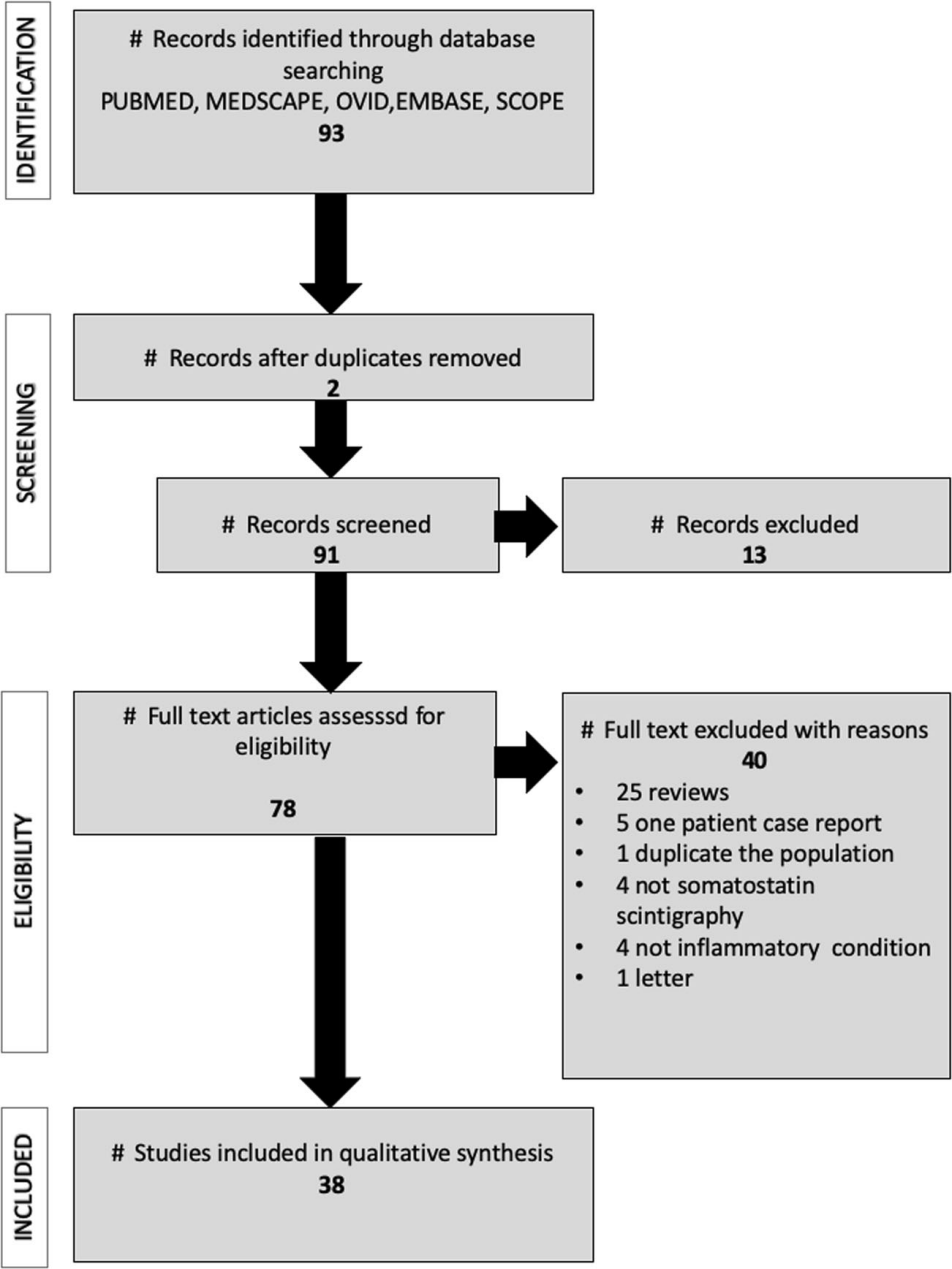
Prismal flowchart of article selection

We found 93 potential studies evaluating radiolabeled SST analogues in chronic inflammatory diseases. After checking for duplicates, 13 papers were immediately excluded; after reading the full-text records, 40 were excluded because they did not fulfill the inclusion criteria. We, therefore, analyzed 38 publications, as shown in Fig. 1. Results are summarized in Table 1.

**Table 1:** Summary of analyzed papers

Author	Type of study	Patients	Radiotracer	Risk of bias	Comments
Endothelial inflammation					
Tarkin 2001^18^	Prospective	42	^18^F-FDG and ^68^Ga-DOTA-TATE	We did not identify source of bias	SRS identifies culprit coronary and carotid arteries in individuals with acute coronary syndrome, TIA and stroke
Malmberg 2015 ^19^	Observational prospective	60	^64^Cu-DOTA-TATE	Reference test: they did not use reference test	SUVmax of inflamed plaques correlates with some Framingham risk factors
Wan 2017 ^21^	Prospective cohort	20	^68^Ga-DOTA-TATE	Flow and timing: Heterogeneity of time between carotid event, PET scan and endarterectomy	No significantly different uptake in carotid plaques and contralateral carotids and no inflammatory cells at histology of excised plaques
Pedersen 2015 ^22^	Prospective cohort	10	^64^Cu-DOTA-TATE	None	Uptake was significantly higher in symptomatic plaque versus the contralateral carotid artery and correlates with CD163 staining of plaques
Xian Li 2012 ^23^	Descriptive retrospective series of cases	16	^18^F-FDG and ^68^Ga-DOTA-TATE	Reference standard and index test: unprecise reference test and lack of standard method for index test analysis.	Correlation between mean uptake of ^18^F-FDG or ^68^Ga-DOTA-TATE and patients’ score of risk factors
Mojtahedi 2014 ^24^	Retrospective series of cases	44	^68^Ga-DOTA-TATE	Reference standard: Inaccurate method	^68^Ga-DOTA-TATE detects more areas with increased uptake in patients with high cardiovascular risk
Rominger 2010 ^25^	Descriptive, retrospective	70	^68^Ga-DOTA-TATE	Reference standard: Inaccurate method.	^68^Ga-DOTA-TATE detects more areas with increased focal uptake in patients with high cardiovascular risk and with calcified plaques
Rheumatoid arthritis					
Anzola 2016 ^30^	Pilot study	18	^99m^Tc-Hynic-TOC	Index test: Lack of standard method for index test analysis.	By ^99m^Tc-Hynic-TOC all patients showed uptake in joints and in 60% of salivary glands. Patients who were evaluated after Infliximab therapy showed significant reduction of joint uptake.
Van Hagen 1994 ^31^	Prospective	18	^111^In-Octreotide	Index test and reference standard: just 2 patients were confirmed with histopathology and lack of standard method for index test analysis.	76% of swollen RA joints were visualized. The degree of pain and swelling correlated well with positive scintigraphy findings in joints
Cardiac allograft rejection					
Aparici 2000 ^61^	Prospective	10	^111^In-octreotide	Index test: lack of standard method for index test analysis.	Preliminary results indicate the feasibility of targeting activated lymphocytes with SRS in the detection of cardiac allograft rejection.
Small vessel vasculitis					
Neumann 2004 ^62^	Pilot study	36	^111^In-octreotide	Reference standard: lack of immunohistochemical analysis to confirm presence of SSTR2 and SSTR3 in the whole population.	^111^In-octreotide for pulmonary disease showed a sensitivity of 86%, specificity of 96% and 97% of positive predictive value for active disease. For ear nose and throat disease 68% and 100%
Idiopathic pulmonary fibrosis					
Ambrosini 2010 ^34^	Prospective	14	^68^Ga-DOTA-NOC	Reference standard index test: it was not possible to confirm the presence of SSTR2 and SSTR3 in the affected areas. Lack of standard method for index test analysis.	^68^Ga-DOTA-NOC uptake corresponded to areas of HRCT abnormalities in IPF patients, supporting the hypothesis that SSTR is over- expressed in lungs of IPF patients.
Lebtahi 2006 ^35^	Prospective	11	^111^In-octreotide	Index test and reference standard: Lack of standard method for index test analysis. Inaccurate reference test.	Increased uptake of ^111^In-Octreotide in (mainly idiopathic) pulmonary fibrosis. Lung uptake correlates with alterations in lung function and with intensity of alveolitis and seems to be related to severity of lung fibrosis.
Win Thida 2012 ^36^	Prospective	26	^18^F-FDG and ^68^Ga-DOTA-TATE	Patient selection, index test, reference standard: Patient heterogeneity of the population, few biopsies just in some of the patients; no threshold defined to interpret the index test.	All patients demonstrated increased pulmonary PET signal with ^68^Ga-DOTA-TATE and ^18−^F-FDG. ^68^Ga-DOTA-TATE might be useful to identify SSTR overexpression in this patient subgroup, and might be used for selecting patients who might benefit from somatostatin analog treatment.
Sarcoidosis and granulomatous diseases					
Lapa 2016 ^51^	Prospective	15	^68^Ga-DOTA-TOC	No source of bias.	Extent of ^68^ Ga DOTATOC PET positive myocardial areas might provide the same prognostic information as shown for ^18^F-FDG-PET/CT in myocardial sarcoidosis. Inflammatory cells.
Kwekkeboom 1998 ^52^	Cross-sectional	46	^111^In-octreotide	Reference test and index test: not all patients had histological proof of granuloma. Lack of standard method for index test analysis.	^111^In-Octreotide somatostatin receptor imaging can demonstrate active granulomatous disease in patients with sarcoidosis.
Lebtahi 2001 ^53^	Prospective	18	^111^In-octreotide and ^67^ gallium	Reference test and index test lack of in vitro analysis of somatostatin receptor presence in lesions. Lack of standard method for index test analysis. Some patients were receiving steroids previous to the scan.	^111^In-octreotide SSTRs seems to be a promising alternative for evaluating the extent of sarcoidosis, detects significantly more sites of sarcoidosis involvement, especially for lung and mediastinal involvement.
Kamphuis 2015 ^54^	Retrospective	175	^111^In-Octreotide	Reference standard and index test: lack of in vitro analysis of somatostatin receptor expression in the lesions. Lack of standard method for index test analysis.	^111^In-Octreotide SRS is additional in the diagnostic workup and more sensitive than conventional imaging in sarcoidosis patients.
Gormsen 2016 ^55^	Pilot study	19	^68^Ga-DOTA-NOC	Patient selection, reference standard, index test: 3 out of 19 patients had confirmed cardiac sarcoidosis; lack of a real reference test to diagnose the cardiac sarcoidosis; variation observer during the analysis of the index test.	^68^Ga-DOTA-NOC can be used as an adjunct imaging modality in patients with suspected cardiac sarcoidosis; preferably as an imaging substitute for the obsolete ^67^Ga-citrate scintigraphy.
Nobashi 2016 ^56^	Retrospective	20	^68^Ga-DOTA-TOC ^67^ Ga	Reference standard: lack of in vitro analysis of SSTR in the lesions.	^68^Ga-DOTA-TOC was superior than ^67^Ga-citrate identifying lymph nodes and in visualizing lesions in the uvea and muscle as well as in the lymph nodes.
Piotrowoski 2012 ^57^	Observational	32	^99m^ Tc-HYNIC-TOC	Reference test and index test: reference test with limitations to detect extrapulmonary disease. Lack of standard method for index test analysis.	Although ^99m^Tc-Hynic-TOC could discriminate between positive and negative studies further studies are needed to find the utility of those results.
Weinmmann 2000 ^58^	Pilot study	13	^111^In-Octreotide	Reference test and index test: not all patients went o biopsy to confirm the activated SSTRs; Lack of standard method for index test analysis.	^111^In-Penteotride identified abnormal uptake in lungs, bone, but not in the skin nor in the liver and central nervous system in patients with granulomatous disease.
Oztürk 1994 ^59^	Descriptive analysis	3	^111^In-octreotide	Descriptive study on three cases.	Descriptive study showed increased uptake of ^111^In-penteotride in granulomatous lesions.
Vanhagen 1994 ^60^	Prospective	20	^111^In-octreotide	Index test: Lack of standard method for index test analysis.	Descriptive study showed increased uptake of ^111^In-Penteotride in granulomatous lesions
Thyroid exophthalmopathy					
Aguirre Balsalobre 2007 ^37^	Series of cases	18	^111^In-Octreotide	Index test: Lack of standard method for index test analysis.	^111^In-Octreotide identified active thyroid orbitopathy. Patients identified as positive, treated with lanreotide showed improvement clinically and scintigraphically.
Colao 1998 ^38^	Series of cases	10	^111^In-Octreotide	Index test: Lack of standard method for index test analysis.	They showed that in a semiquantitative analysis with ^111^In-pentreteotide for patients with higher scores in the orbital uptake, it is possible to predict the therapeutical outcome in these patients.
Burggasser 2003 ^47^	Observational prospective	44	^99m^TcP829	Index test: Lack of standard method for index test analysis.	Orbital uptake ratios were significantly different between patients with active and non-active orbitopathy. A statistically significant correlation was found between the CAS of the orbital disease and ^99m^TcP829 tracer uptake.
Gerdin 1999 ^39^	Prospective observational	22	^111^In-Octreotide	Index test: Lack of standard method for index test analysis.	Quantitative measurement of orbital ^111^Octreotide uptake might be of use in predicting the outcome of immunosuppressive and radiotherapy treatment of patients with Graves ophthalmopathy.
Huan Sun 2007 ^48^	Prospective observational	14	^99m^Tc-Hynic-TOC	Index test: Lack of standard method for index test analysis.	Orbital ^99m^Tc-Hynic-TOC can be useful for the estimation of disease activity and prediction of the response to radiotherapy in Graves’ ophthalmopathy patients
Kahaly 1998 ^40^	Prospective observational	20	^111^In-octreotide	Index test: lack of standard method for index test analysis.	^111^In-octreotide was a sensitive technique with a high positive predictive value to select those patients who might benefit from treatment with immunosuppressive agents.
Kahaly 1995 ^41^	Prospective observational	44	^111^In-Octreotide	Index test: Lack of standard method for index test analysis.	Graves’ ophthalmopathy patients showed markedly orbital accumulation of ^111^In-pentetreotide in contrast to controls.
Krassas 1999 ^42^	Prospective	14	^111^In-Octreotide	Index test: Lack of standard method for index test analysis.	^111^In-Octreoscan correlates well with the clinical activity of the thyroid exophthalmopathy and orbital accumulation of radioactivity was diminished after treatment with somatostatin analogues.
Krassas 1995 ^43^	Prospective	20	^111^In-Octreotide	Index test: Lack of standard method for index test analysis.	^111^In-Octreoscan could predict those patients with Graves’ thyroid eye disease who might benefit from treatment.
Lincke 2009 ^50^	Prospective	73	^111^In-pentetreotide and ^68^Ga-DOTA-TOC	Index test, reference standard: lack of standard method for index test analysis; there was not immunohistochemical confirmation of activated SSTRs in the thyroid nodules.	Normal thyroid tissue shows detectable ^111^In-Pentetreotide and ^68^Ga-DOTA-TOC which could indicate a basal SSTR expression in normal tissue. Hot nodules showed increased uptake of both tracers also for active Hashimoto and Graves.
Moncayo 1999 ^44^	Prospective	51	^111^In-octreotide	Index test: lack of standard method for index test analysis.	Positive ^111^In-Octreotide in patients Images provide useful information on the efficacy of immunosuppressive therapy.
Nocaudi 1999 ^45^	Prospective	17	^111^In-Octreotide	Index test: lack of standard method for index test analysis.	^111^In-pentreteotide scintigraphy may be a good indicator of the likelihood of evolution in thyroid-associated ophthalmopathies.
Postema 1994 ^46^	Cross sectional	58	^111^In-Octreotide	Patient selection, index test: lack of standard method for index test analysis.	Thyroidal and orbital Graves’ disease can be visualized by ^111^In-Octreotide reflecting disease activity.
Rong Zhao 2012 ^49^	Prospective	46	^99m^Tc-Hynic-TOC	Index test: lack of standard method for index test analysis.	Orbital 99mTc-TOC fusion imaging is able to determine the pathological phase of Graves’ disease, giving a high positive scan in the active early phase and a low positive or negative scan in the stable end phase of the disease.

### Endothelial inflammation and atherosclerosis

In total, we analyzed 9 studies and excluded 2 for the analysis because they did not complete the inclusion criteria; 7 studies with the use of SRS for endothelial inflammation were included, with a total of 262 patients (Table 1). In details, Tarkin et al. [18], in a descriptive study, compared PET with ^18^F-fluorodeoxyglucose (FDG) and ^68^Ga-DOTA-TATE to detect culprit coronary and carotid arteries with acute coronary syndrome, transient ischemic attacks and stroke, and demonstrated best results with ^68^Ga-DOTA-TATE in the diagnostic accuracy to detect stable and unstable inflamed coronary lesions and vascular inflammation in neighboring coronary and aortic vasculature. When age, total cholesterol, and BMI were evaluated with other relevant clinical factors using multi- variate linear regression, they remained significant predictors of ^68^Ga-DOTATATE mTBRmax. In patients with TIA or stroke, increased ^68^Ga-DOTATATE inflammatory signals were reliably differentiated between culprit carotid plaques and contralateral nonculprit carotid arteries. Ex vivo ^68^Ga-DOTATATE carotid autoradiography showed high levels of specific ligand binding to SSTR2 receptors. SSTR2 and CD68 mRNA levels were highly correlated within carotid plaque. Carotid SSTR2 and CD68 mRNA levels also showed strong correlation with in vivo ^68^Ga-DOTATATE TBRmax values measured at the corresponding level in clinical PET images. These data provided both histological and molecular validation of ^68^Ga-DOTATATE as specific marker of atherosclerotic inflammation. Under QUADAS analysis, we did not find any significant source of risk bias in this report. Malmberg et al. [19] in an observational study in 60 consecutive patients described the differences between ^68^Ga-DOTA-TOC and ^64^Cu-DOTA-TATE in detecting atherosclerotic plaques in different vessels, showing better performance of ^64^Cu-DOTA-TATE explained by the longer half-life which allows for early as well as late PET scanning, even the day after injection. ^64^Cu has also a substantially shorter positron range than ^68^Ga, 1 versus 4 mm, rendering it a much better spatial resolution, but a lower positron abundance [20] Uptake of ^64^Cu-DOTATATE was significantly higher than ^68^Ga-DOTATOC in the vascular regions both when calculated as maximum and mean uptake. In a multivariate analysis, there was a significant association between Framingham risk score and the overall maximum uptake of ^64^Cu-DOTATATE using SUV as well as target-to-background ratio, whereas no association was found with ^68^Ga-DOTATOC. The association of risk factors and maximum SUV of ^64^Cu-DOTATATE was found driven by body mass index, smoking, diabetes, and coronary calcium score suggesting the potential use of this radiotracer as noninvasive biomarker of cardiovascular risk. According to QUADAS 2 analysis, we considered the reference test as the main source of risk of bias because it did not use the anatomopathological evidence to validate the results on 68Ga-DOTATOC but the calcium score on CT. Simon Wan et al. [21] conducted a descriptive study with 20 patients with recent stenotic carotid events and described the findings of ^68^Ga-DOTA-TATE in carotid plaques versus histology. Surprisingly, they did not find any significant uptake of ^68^GaDOTATOC in the plaques neither activated macrophages expressing SSTR2; and they could not support the use of ^68^GaDOTATOC for evaluating the inflamed plaque; the source of bias in this report, (misclassification explained by heterogeneity of time between carotid event, research PET, and endarterectomy) could maybe explain the results. In QUADAS 2 analysis, this source of bias corresponds to flow and timing. Pedersen et al. [22], in a population of 10 patients with clinical symptoms of stroke or transient ischemic attack, described the findings of ^64^Cu-DOTA-TATE uptake in atherosclerotic plaques. ^64^Cu-DOTA-TATE uptake was significantly higher in symptomatic plaques and correlated with CD163 expression by activated macrophages at histology. We did not find any important source of risk of bias in this report according to QUADAS analysis. Xian Li et al. [23] in a retrospective series of 16 patients, analyzed simultaneously ^18^F-FDG and ^68^Ga-DOTA-TATE PET/CT and showed significantly increased uptake in the fibrotic and vulnerable atherosclerotic plaques compared with normal coronary arteries, suggesting a potential role of this tracer for molecular assessment of coronary artery disease. They found higher values of focal uptake with ^68^GaDOTATATE in patients with high cardiovascular risk and significant correlation between the mean uptake of both tracers and the patient’s score of risk factor. We found that the reference test and the index test were source of risk of bias in QUADAS 2 analysis. Mojtahedi et al. [24] generated a preliminary data by using ^68^Ga-DOTATATE PET/CT, with in vulnerable or fibrotic atherosclerotic plaques in the coronary arteries. In a population of 44 patients with neuroendocrine tumors (NET) who underwent ^68^Ga-DOTATATE PET/CT, they found that the mean TBR value in the normal group was 1.345 ± 0.58 while the mean TBR value in the fibrotic plaque group was 1.752 ± 1.50 (*p* 0.0043) and in atherosclerotic plaques group was 2.043 ± 1.76, *p* < 0.0001). There was a significant correlation (*p* = 0.0026) between ^68^Ga-DOTATATE uptake and the progression to formation of atherosclerotic plaques, based on coronary CT calcium score, HU. Those findings suggested the potential of ^68^Ga-DOTATATE PET/C for molecular assessment of coronary artery disease. According to QUADAS 2 analysis, we considered as potential source of risk bias the reference standard they used for validate the ^68^Ga-DOTATATE PET results. Rominger et al. [25] in a descriptive analysis of 70 consecutive patients correlated ^68^Ga-DOTATATE uptake in the left anterior descending coronary artery with the presence of calcified plaques and cardiovascular risk factors. Higher uptake lead distinguishes between patients with and without coronary calcifications. As in the abovementioned reports, the main source of bias of this series in QUADAS 2 analysis was related to the reference standard they used to validate the ^68^Ga-DOTATATE results.

It is well known that monocytes and macrophages, being the first inflammatory cells associated with vascular inflammation, play an important role in the pathogenesis of atherosclerosis, contributing to necrotic core formation, fibrous cap thinning, and plaque vulnerability [26]. These cells express SSTR-2 on the cell surface in cultures [27] and therefore ^68^Ga-DOTA-TATE uptake was thought to have the potential to be a surrogate marker of inflammation to study plaque biology [28]. Taking together all published data, it is likely that there are complex relationships between the stage of plaque evolution (chronic vs acute), macrophage density and activity, SSTR subtype expression, and ^18^F-FDG and ^68^Ga-DOTA-TATE uptake. Evidences, so far, show a tendency to confirm the utility of SRS with ^68^Ga-DOTA-TOC to detect high-risk, vulnerable, atherosclerotic plaques and an interesting correlation with risk factors. Added value of TOC (specific for inflammatory cells only provides an in vivo information on the vulnerability of plaques) vs FDG (global inflammatory burden but non-specific and does not provide a relevant histopathological information) and ^18^F-Na and calcium score [29].

### Rheumatoid arthritis

In total, 11 publications were analyzed, but just 2 clinical studies were included within 36 patients (Table 1), since most were reviews, case reports or in vitro or animal studies. Anzola et al. [30] conducted a pilot study to evaluate the uptake pattern of ^99m^Tc-EDDA/HYNIC-TOC in the joints of 18 patients with rheumatoid arthritis refractory to treatment. They showed high uptake of the molecule in all evaluated joints and also in the salivary glands of some patients as possible evidence of Sjogren syndrome. There was a statistically significant reduction in uptake after treatment with anti-TNFα antibodies in all patients. The authors concluded that this scan can be useful to select and monitor RA patients for therapy with anti-TNFα antibodies. In QUADAS 2 analysis, we found as potential source of risk of bias the index test because the lack of standardized method to evaluate the results. Vanhagen et al. [31] reported the results of 14 patients with RA and 4 with osteoarthritis using ^111^In-octreotide. They reported sensitivity (uptake of the tracer in swollen and painful joints) of 76% in RA patients and of 20% in osteoarthritis. As in the abovementioned series, we found lack of a standardized method to evaluate the results and weakness in the reference test used to compare the results (QUADAS 2, reference and index test source of bias).

Synovia of patients with active rheumatoid arthritis expresses a high density SSTR [32]. The experience published in these two clinical papers, shows the potential of SRS to localize and identify sites of active inflammation in joints, both symptomatic and asymptomatic, and extra-articular involvement as in salivary glands. The information provided by SRS is different from other imaging modalities and provides in vivo histopathological information on the activity of cell-mediated inflammation. This information has an important impact for therapy decision-making and therapy follow-up as also appears in other studies [33].

### Idiopathic pulmonary fibrosis

In total, 7 studies were analyzed, but only 3 studies could be included within 51 patients (Table 1). Ambrossini et al. [34] in an observational study evaluated the potential role of ^68^Ga-DOTANOC PET/CT in patients with idiopathic pulmonary fibrosis (IPF) and non-specific interstitial pulmonary pneumonia (NSIP). ^68^Ga-DOTANOC PET/CT findings were compared with HRCT results. In IPF patients, ^68^Ga-DOTA-NOC uptake appeared as a typical subpleural and peripheral distribution involving both lung fields predominantly at the bases. Areas of ^68^Ga-DOTA-NOC uptake directly corresponded to pathologic areas on HRCT. In contrast, ^68^Ga-DOTANOC uptake was faint in NSIP patients and undetectable in healthy control subjects. No significant association was documented in NSIP cases. According to QUADAS 2 analysis, we found as source of potential risk of bias the index test because they did not use a previous standardized method to evaluate the results and they did not confirm the findings under histopathogical analysis.

Lebtahi et al. [35] investigated the expression in vivo of SSTR in the lungs of 11 patients with IPF, and 6 patients with pulmonary fibrosis associated with systemic sclerosis by using ^111^In-octreotide scintigraphy and compared the results with 19 control patients. They reported higher uptake in all 11 IPF patients and in 4 of 6 systemic sclerosis patients related to normal control population. Increased uptake correlated significantly with different lung function and cytological tests. As in the abovementioned series, according to QUADAS 2 analysis, we found as potential source of risk of bias the index test and the reference test explained by the same conditions argued before.

Win Thida et al. [36] analyzed in an observational study 26 patients with diffuse parenchymal lung disease (10 patients with idiopathic pulmonary fibrosis, 12 with nonspecific interstitial pneumonia and 4 with other forms of interstitial lung disease), by using ^68^Ga-DOTA-TATE and ^18^F-FDG by combined PET and HRCT. All patients demonstrated increased pulmonary signal with both ^68^Ga-DOTA-TATE and ^18−^F-FDG.

The experience reported in this review shows the potential utility of the SSTR scintigraphy to localize and to detect the active inflammation mediated by an immunological response in idiopathic pulmonary fibrosis. In QUADAS 2 analysis, we found as source of risk of bias the reference test and index test explained by the same reasons argued above.

### Graves’ ophthalmopathy

**Fig. 2:**
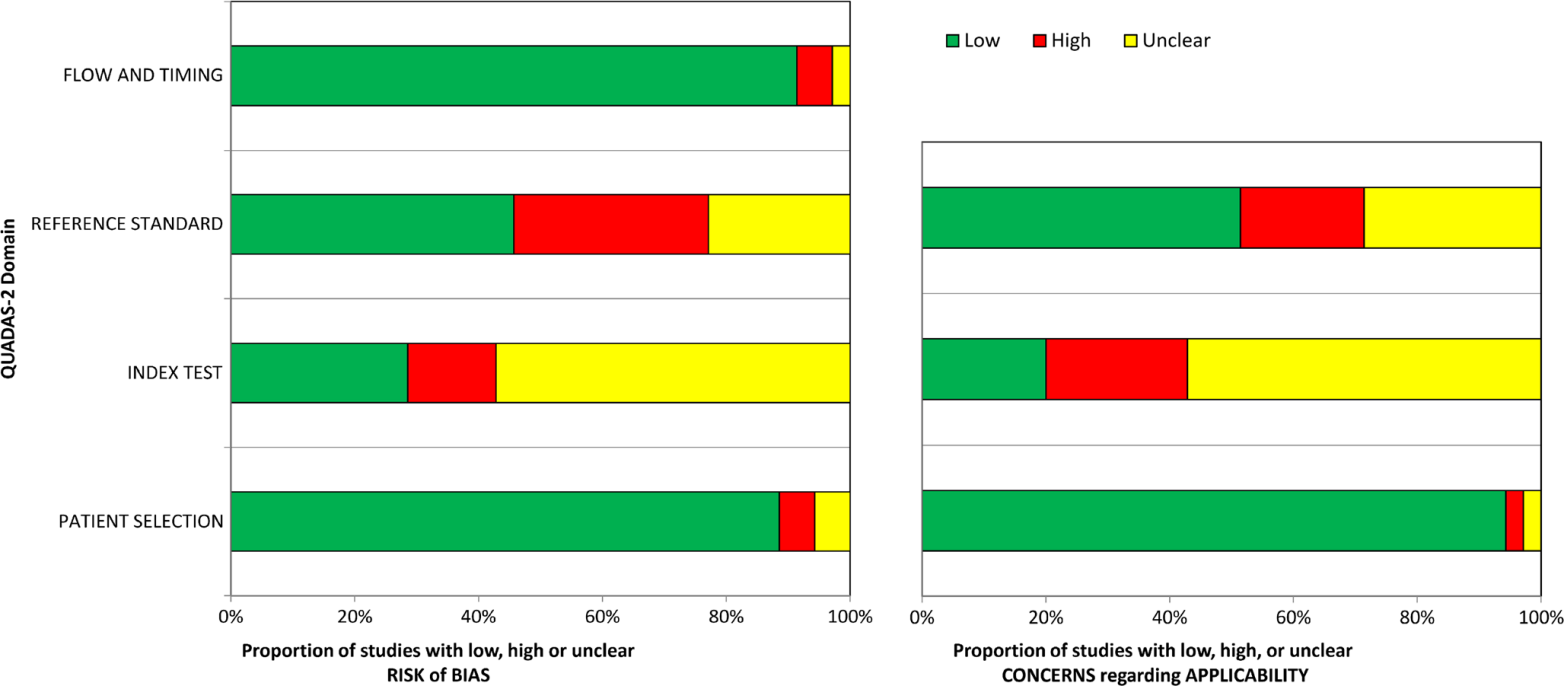
Graphical representation of frequencies of biases in analyzed papers by QUADAS 2

In total, 33 papers were reviewed, 14 studies with in total 451 patients were included (Table 1). Aguirre-Balsalobre et al. [37] in a series of 18 patients affected by thyroid orbitopathy with Graves’ diseases by using a semiquantitative analysis of the images with ^111^In-pentreotide, demonstrated how patients with higher scores had better response to corticosteroid therapy; according to QUADAS 2, analyzing the source of bias of this experience was related to the index test because lack of standardized method. Colao et al. [38], Gerdin et al. [39], Kahaly et al. [40], Kahaly et al. [41], Krassas et al. [42], Krassas et al. [43], Moncayo et al. [44], Nocaudie et al. [45], Postema et al. [46], in similar study designs by using ^111^In octreotide, described how patients with active orbitopathy in Graves’ disease had higher uptake of the tracer in the orbits, and how this finding was helpful to discriminate active disease Vs non-active disease and how it helped to choose better the candidates to immunotherapy, radiotherapy or somatostatin analogues therapy. In those series, it was possible to observe how patients with higher uptake in orbits responded well to the installed treatment. The common source of bias for the abovementioned authors according to QUADAS 2 analysis was related to the index test, and although none of the authors used a standardized method to analyze the images, the results were not only similar but also promissory; moreover, in Postema’s report, it was seen with a high frequency of source of risk bias related to patient selection. In Postema’s series, it also found the patient selection as source of risk of bias (Fig. 2).

Burggasser et al. [47] in a series of 44 consecutive patients, by using ^99m^TcP-829, demonstrated statistically the differences in the orbital uptake between control and disease patients and between active and inactive orbitopathy, in Graves’ disease patients with orbitopathy. Even though they used two different methods to analyze the images, we considered the index test as source of bias according to QUADAS 2 analysis. Huang Sung et al. [48], by using ^99m^Tc HYNIC-TOC, reported similar results of the above authors with the same previous described source of bias.

Rong Zhao et al. in a prospective study [49] by using SPECT/CT system as novel technique, investigated the predictive role of the orbital somatostatin receptor scintigraphy with ^99m^Tc-EDDA/HYNIC-TOC (^99m^Tc-TOC) to detect clinical stage of Graves’ ophthalmopathy (GO) and the response to corticosteroid therapy in a sample of 46 patients with GO and four volunteers without eye disease. The treatment effect was evaluated both by the orbital ^99m^Tc-TOC uptake and NOSPECS. Orbital images were quantified by region of interest (ROI) analysis. Semi-quantitative evaluation of retrobulbar uptake was performed with irregular ROIs which were placed over the orbits (O) and the reference area over the occipital area (OC). This prospective study demonstrated marked orbital accumulation of ^99m^ Tc-TOC in 22 GO patients, showing higher activity score and clinical improvement; they demonstrated good correlation of orbital ^99m^ Tc-TOC scintigraphy with CAS. The value of this study was to identify the anatomical structures depicted with ^99m^ Tc-TOC in patients with GO by means of SPECT and CT image fusion analysis. Thus, in the absence of histology, because of a favorable target-to-background ratio and fusion imaging of SPECT/CT in this study, orbital 99mTc-TOC fusion imaging was able to determine the pathological phase of Graves’ disease, giving a high positive scan in the active early phase and a low positive or negative scan in the stable end phase of the disease. We consider that the analysis of the images under hybrid images diminishes the risk of bias related in index test (QUADAS 2).

Lincke et al. [50] is the only report so far that used PET study in thyroid-related pathologies. He compared the characteristics of uptake between ^111^In-pentreotide and ^68^Ga-DOTA-TOC in normal and pathologically altered thyroid glands and found that both tracers showed higher uptake in active Hashimoto and Graves’ disease most likely caused by SSTR expressing lymphocytes in the thyroid tissue; however, the physiologic or pathophysiologic relevance of the increased In-111 pentetreotide and Ga-68 DOTATOC uptake in normal thyroid glands, hot and cold nodules, and goiters and nodular thyroids remain to be determined. According to QUADAS 2 analysis in this report, the index test and the reference test were considered as unclear sources of bias.

During the last 20 years, it demonstrated the utility of SSTRs scintigraphy identifying active orbitopathy in Graves’ exophthalmopathy. No matter as a common source of bias in those series it was found to be lacking of validated method to evaluate the SSTR scintigraphies, all the authors reported similar results showing scintigraphic patterns consistently with high uptake of the molecule in the periorbitary zone as evidence of active disease with prognostic implications.

### Sarcoidosis and other chronic inflammatory diseases

**Fig. 3:**
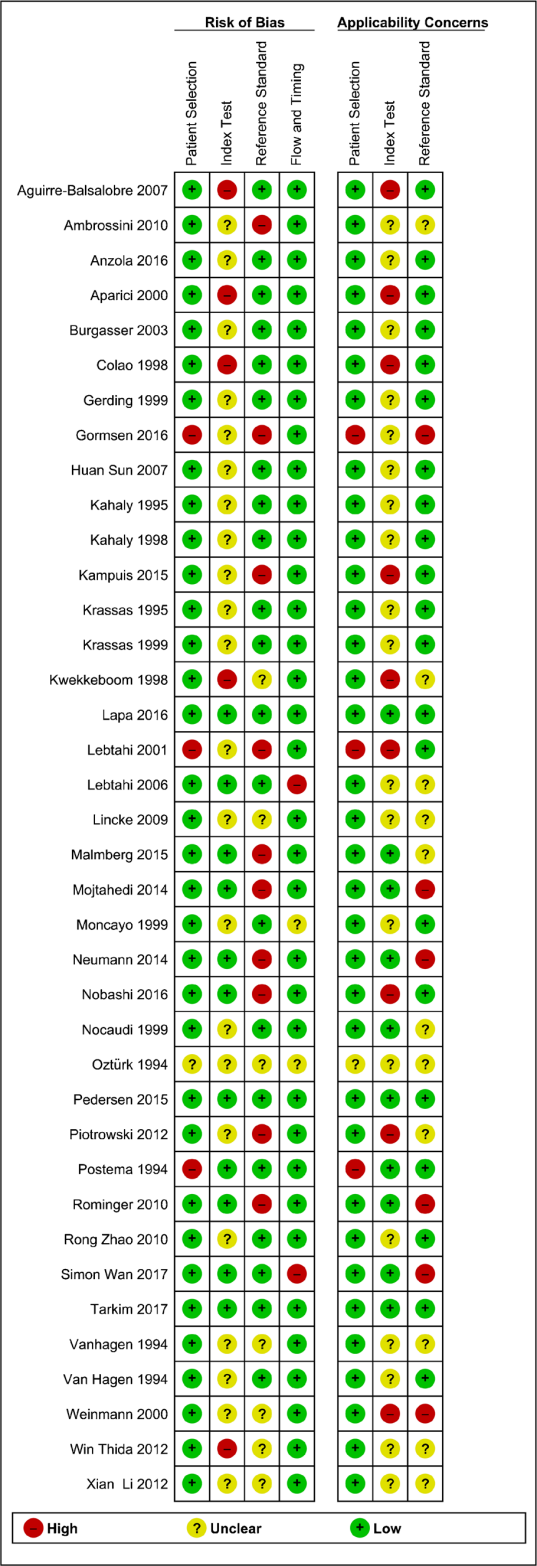
Graphical representation of biases in each analyzed papers by QUADAS 2

As far as sarcoidosis is concerned, were reviewed 18 publications, of which 6 were excluded (Table 1). Lapa et al. [51] in an observational study conducted a pilot prospective study in 6 patients with active peri−/myocarditis and 6 with sub-acute myocardial infarction, who underwent SSTR-PET/CT, ^68^Ga DOTA-TOC and cardiac MRI within 3–10 days after onset of symptoms. They compared the results under semiquantitative analysis with 12 oncologic patients with no history of coronary artery disease. In patients with clinical evidence of myocarditis, PET/CT returned 26 positive segments and MRI 13 segments, respectively. In patients with myocardial infarction, there were 29 ^68^Ga-DOTA-TOC positive segments and 31 abnormal segments in MRI. On a head-to-head comparison, SSTR-PET and MRI were concordantly positive in 36 segments (76.6%). Nineteen segments were SSTR-PET positive and MRI-negative (9.3%; 19/204), and 11 SSTR-PET negative and MRI-positive (5.4%; 11/204). Both modalities returned negative results in 138 segments, thus leading to an overall concordance of 85.3%. Agreement of the modalities was higher in patients with myocardial infarctions. SUV mean and SUV max were significantly higher in the infarcted/ inflamed myocardium as compared with remote myocardium or the left ventricular (LV) cavity. We did not find any relevant risk of bias according to QUADAS 2 analysis (Fig. 3). The overall concordance of the 2 modalities was 96.1%. Kwekkeboom et al. [52] in a cross sectional study in 46 patients with known mediastinal, hilar, and interstitial sarcoidosis, by using SRS with ^111^In-pentetreotide showed positive results in 36 out of 37 patients with known pathology, and in 7 with normal X-rays. In 13 patients who had no evidence of extrapulmonary disease involvement with physical examination and conventional imaging, somatostatin receptor imaging revealed abnormal uptake of radioactivity outside the chest. Neither the degree of radioactive accumulation nor a specific pattern of pathological uptake was correlated with disease severity or clinical course. They repeated the scintigraphy on 13 patients, 5 of 6 patients who showed X-rays improvement, the SSTRs also improved. Two of 5 patients whose X-rays did not improve, lung function and SSTRs improved. They could not conclude that pentetreotide scintigraphy has a role in predicting prognosis or clinical course in patients with sarcoidosis. According to QUADAS 2 analysis, the source of risk of bias in this series was related to the lack of a standardized method to evaluate the results in the index test, and also we found a low concern related to the reference test used in some situations. Lebtahi et al. [53] in an observational study demonstrated the superiority of ^111^In pentetreotide compared with ^67^Ga scintigraphy in the evaluation of pulmonary and extrapulmonary involvement in 18 patients with proven sarcoidosis especially in the hilar or mediastinal area. Nine were or recently had been receiving steroid therapy at the time of the examination. Gallium scintigraphy found abnormalities in 89% of patients and detected 65% of the clinically involved sites. SRS found abnormalities in 100% of patients and detected 83% of clinically involved sites. Of the 9 treated patients, gallium scintigraphy found abnormalities in 78%, detecting 59% of the clinically involved sites whereas SRS found abnormalities in 100%, detecting 82% of the clinically involved sites. SRS images were consistently better than gallium images, with well-delineated lesions, especially in the hilar or mediastinal area, on either planar or SPECT images. They concluded that SSTRs seems to be a promising alternative to gallium scintigraphy for evaluating the extent of sarcoidosis, detects significantly more sites of sarcoidosis involvement with lesion contrast significantly higher than with gallium scintigraphy, especially for lung and mediastinal involvement. Despite the good results reported, we found as potential source of bias in QUADAS 2 analysis the lack of standardized method to evaluate the images on ^111^In pentetreotide and also the lack of a well-defined reference test. Kamphuis et al. [54] in a retrospective study evaluated the additive value of SRS scintigraphy with ^111^In pentetreotide in the clinical evaluation of sarcoidosis and compared the results with chest X-ray and CT. In both histologically proven and unproven sarcoidosis, in all but one SSTR uptake was demonstrated. In the thoracic region SRS increased the yield with 36% and 32% in comparison with X-ray and CT, respectively. In the histologically proven group, there were no negative SRS results, and the SRS increased the yield for thoracic localization in 30% and 14% of the patients in comparison with X-ray and CT, respectively. As in the abovementioned series, although the results reported were encouraging, we found in QUADAS 2 analysis that the lack of a standardized method for evaluating the SRS scintigraphy with ^111^In pentetreotide was a source of potential bias as well as the reference test was source of potential risk of bias. Gormsen et al. [55]in a pilot study in 19 patients with suspected cardiac sarcoidosis compared the diagnostic accuracy and inter-observer variability of ^68^Ga-DOTANOC vs ^18^F-FDG PET/CT. Cardiac sarcoidosis (CS) was diagnosed in 3/19 patients. By consensus, 11/19 ^18^F-FDG scans were rated as inconclusive, resulting in low agreement among reviewers (Fleiss combined kappa 0.27) and correspondingly poor diagnostic accuracy; for ^68^Ga-DOTANOC, no scan was rated as inconclusive but it was reported as a poor interobserver agreement (Fleiss combined kappa 0.46). The sensitivity of ^18^F-FDG PET for diagnosing CS was 33%, specificity was 88%, PPV was 33%, NPV was 88%, with 79% of diagnostic accuracy; in contrast to ^68^Ga-DOTANOC, and accuracy was 100%. ^68^Ga-DOTANOC PET/CT looks very promising as an alternative CS PET tracer. When we analyzed under QUADAS 2 protocol, we found in the index test and reference test the potential source of risk of bias.

Nobashi et al. [56] in a descriptive study compared the utility of ^68^Ga-DOTA-TOC with conventional ^67^Ga-citrate in the visualization of active sarcoidosis and correlated quantitative parameters on ^68^Ga-DOTA-TOC PET/CT with clinical data. Twenty patients with sarcoidosis underwent both studies. The diagnosis was confirmed in 12 patients histologically and in the other 8 on clinical and paraclinical data. Active lesions were identified by visual interpretation when focal increases in radioactivity were higher than the normal biodistribution of tracers. Quantitative analysis on ^68^Ga-DOTATOC was made by using SUVmax values. DOTATOC-PET/CT showed abnormal findings in 19 patients, whereas ^67^Ga showed abnormal findings in 17. DOTATOC was superior than ^67^Ga identifying lymph nodes (*p* < 0.046). SUVmax in each organ did not show statistical difference between involved organs; however, the average SUVmax of lymph nodes was highest among the involved organs. It was not seen as statistically different in SUVmax between patients with and without symptoms. Active lesion volume calculated by DOTATOC-PET moderately correlated with serum ACE concentrations. ^68^ GaDOTATOC-PET was superior to ^67^Ga in visualizing lesions in the uvea and muscle as well in the lymph nodes. As a source of bias, there was partial verification bias because the lack of in vitro analysis of somatostatin receptor in the lesions and because not all patients were diagnosed by the same reference standard. There is patient variation bias in some patients who were receiving treatment for the clinical condition in the same time when there were acquired studies. According to QUADAS 2 analysis, the potential source of bias in this series relied on the index test and reference standard explained by the same arguments in the above references.

Piotrowoski et al. [57], in an observational study, evaluated 32 patients with sarcoidosis and used ^99m^Tc-HYNIC-TOC scintigraphy as a reference method to evaluate the clinical usefulness of traditional markers, such as angiotensin-converting enzyme (SACE), C-reactive protein (CRP), serum calcium (S-Ca^2+^ level, and 24-h Urinary-Ca^2+^ los, bronchoalveolar lavage fluid (BALF lymphocytes) as well as of a novel marker of lipid peroxidation-8-IP in exhaled breath condensate (EBC 8-IP). They divided the patients according to the ^99m^Tc-HYNIC-TOC results in two groups: grade 1 (20 patients) when the scintigraphy was positive for an abnormal uptake in thorax and grade 0 (12 patients) when the scan was negative. The only significant difference between scintigraphic negative vs positive results was found in the level of EBC 8-IP; unfortunately, this marker is not specific for sarcoidosis. Furthermore, it observed a trend towards higher levels of SACE in the group with positive radiotracer uptake. Although the tendency was noticed towards higher percentage in patients with positive scintigraphy results, the statistical significance was not reached. It was not possible to demonstrate the capacity of any laboratory test to estimate the intensity of the inflammatory process. The SSRTs could discriminate between positive and negative studies but further studies are needed to find the utility of those results. The reference standard was the most frequent source of bias and applicability concern in QUADAS analysis.

SRS has been proposed also for studying other chronic inflammatory conditions, in particular in histiocytosis, tuberculosis, cardiac allograft rejection, and small vessel vasculitis. Weinmann et al. [58] evaluated 13 patients with histiocytosis. Lung uptake of ^111^In-pentreotride visually and semiquantitative analyzed was statistically significantly higher in patients compared with controls. This report showed concern as potential source of bias in the reference and index test. Oztürk et al. [59] described three similar cases: 2 patients with pulmonary sarcoidosis and one with tuberculosis by ^111^In-pentreotide scintigraphy; this report generated in QUADAS 2 analysis concern about the source of risk of bias in the three items contained in the evaluation: patient selection, index test, and reference standard. Vanhagen et al. [60] reported in a consecutive series of 20 patients the utility of ^111^In-octreotide localizing sites of granuloma infiltration in patients with sarcoidosis and tuberculosis. In vitro autoradiography of fresh tissue biopsies showed bonding of the molecule at sites that were microscopically identified as granulomatous disease. The major contribution of this study was that this scintigraphy procedure demonstrated the expression of somatostatin receptor in such kind of granulomas. Under QUADAS 2 analysis, we found index test as a potential risk of bias because of the lack of a standardized method to evaluate the results.

Aparici et al. [61] conducted a prospective study in 13 patients with suspected cardiac allograft rejection to assess the feasibility of SRS with ^111^In-pentreotide to target activated lymphocytes in transplanted heart as a possible early marker of rejection. A high cardiac uptake was observed in 8 patients: 3 had an acute rejection and 5 had mild or no rejection. However, in 4 of the 5 patients with no rejection at time of study, a biopsy, performed 1 week later, demonstrated a significant rejection requiring treatment. These preliminary results indicate the feasibility of targeting activated lymphocytes for the detection of cardiac allograft rejection and suggest a possible predictive role of SRS. These preliminary results indicate the feasibility of targeting activated lymphocytes with somatostatin receptor imaging in the detection of cardiac allograft rejection.

Index test generated in QUADAS analysis the high concern as source of bias and eligibility concern. The method used to evaluate the pattern uptake was not validated previously.

Neumann et al. [62], by using 111 In-pentreotide, analyzed 32 consecutive patients with ANCA-associated small vessel disease (AASV). Disease activity was evaluated with the Birmingham Vasculitis Activity Score (BVAS). For pulmonary AASV, SRS showed a sensitivity of 86% and a specificity of 96% with a positive predictive value of 97% for active disease. False negative scans were seen in patients under immunosuppressive therapy. In patients with ear/nose/throat involvement, SRS showed a sensitivity of 68% and a specificity of 100% with a positive predictive value of 100%. In patients who responded to therapy and went into full remission, repeat SSTR scintigraphy demonstrated the absence of previously present tracer accumulation; patients with aggressive disease who responded poorly to immunosuppressive therapy remained positive at repeat scintigraphy. Immunohistochemical analysis for SSTR2 and SSTR3 was performed on three open lung biopsies in active disease obtained from two patients with Wegener’s granulomatosis (WG) and one patient with microscopic polyangiitis as well as on nasal and lung tissue of one autopsy case with WG. All specimens demonstrated STR2 y and STR3 expression on monocytes-macrophages and giant cells surrounding granulomas and occasionally in the center of the granulomatous reactions. The reference standard generated in QUADAS analysis high concern as source of bias and applicability concern, because the immunohistochemical analysis was obtained just in three patients.

Reported evidence so far shows the utility of ^68^Ga-DOTA-TOC for detecting sarcoidosis lesions, especially for lymph nodes, uvea, and muscles, as well as for vasculitis and other chronic inflammatory diseases. In particular, the results obtained for monitoring cardiac allografts open a new important clinical application of SRS to early detect lymphocytic infiltration in transplanted tissues and to monitor the effect of therapies in preventing rejection.

## Discussion

This is the first systematic review performed about the usefulness of SRS (both SPECT and PET) to diagnose chronic inflammatory diseases. The role of somatostatin as mediator in inflammatory processes and how different immunological cells express SSTRs is well known. The possibility to specifically target the SSTRs by molecular imaging gave us the opportunity to visualize active inflammation in a variety of inflammatory disorders.

In general, based on the results of this systematic review, we found that SRS has a large potential to be used as diagnostic tool in patients suspected to have chronic inflammatory diseases. The evidence reported until now is supported mainly by observational studies that were designed to investigate different groups of chronic inflammatory conditions: we found a wide heterogeneity in used protocols, in studied conditions, and in the studied population. Furthermore, we observed that in almost every study a validated and standard method to analyze the images was lacking, a condition that became the most important source of bias.

The most frequent pathology evaluated by PET was the endothelial inflammation and vulnerable plaques, where promising correlations between quantitative uptake and histopathology were found, emphasizing the role of SRS for this inflammatory condition. Monocytes and macrophages were the first inflammatory cells to be associated with atherosclerosis. They are believed to play important roles in its pathogenesis, contributing to necrotic core formation and fibrous cap thinning in advanced atherosclerosis, features thought to confer vulnerability [26] It is known that human macrophages express sst2 on their cell surface in cell culture [27]. ^68^Ga-DOTATATE, a specific sst2 receptor agonist, therefore was thought to have the potential to be a surrogate marker of inflammation to study plaque biology [28]. Taken together, it is likely that there are complex relationships between stage of plaque evolution (chronic vs. acute), macrophage density and activity, somatostatin receptor subtype expression, ^18^F-FDG, and somatostatin receptor PET signal. The today evidence shows a high tendency to confirm the utility of the SSTRs ^68^GaDOTATOC to detect the high risk atherosclerotic plaque and its interesting correlation with risk factors. Studies are needed to confirm those finding with better designs which enables to choose better the study population, to calculate a good sample size and to validate the findings with good references tests.

The peripheral nervous system and its neuropeptidergic pathways may play an important role in the pathogenesis and development of rheumatoid arthritis. Somatostatin via its receptors has shown to be implicated in inflammatory diseases and particularly it is known how the synovium in active rheumatoid arthritis express a high density of somatostatin receptors [32]. The experience published in these papers although lacks of a rigorous methodology, shows the potential of radiolabeled somatostatin receptors to localize and identify sites of active inflammation in joints and extra articular involvement and to identify the patients who could benefit of the therapy. In this setting it will be necessary to conduct rigorous prospective studies to validate the methodology to make an accurate approach in arthritis patients.

Different radiotracers were used in patients with Idiopathic Pulmonary Fibrosis (IPF). Ambrosini reported results with ^68^Ga-DOTANOC, which presents the broader SSTR-subtype affinity [63], a favorable dosimetry, and no uptake in the intact lung [64]; recent preclinical evidence demonstrated SSTR expression on fibroblasts of both murine models of IPF and human tissue samples from IPF patients [65]. Although ^68^Ga-DOTANOC binding sites within the lungs cannot be precisely localized without direct sampling, the evidence that tracer uptake was observed in IPF cases allows one to speculate that PET with ^68^Ga-DOTANOC might be useful to identify SSTR overexpression in this patient subgroup. In Ambrosini’s report areas of ^68^Ga-DOTANOC uptake directly corresponded to pathologic areas on HRCT and the preliminary data supported the hypothesis that SSTR is over expressed in the lungs of IPF patients. Lebtahi showed an interesting correlation between STRR scintigraphy findings with 111In-Octreotide in IPF patients and functional pulmonary tests. Win Thida reported results with 68Ga-DOTATATE and 18F-FDG with similar pattern of distribution with both tracers. The experience above mentioned, although has methodological deficiencies showed clearly the potential utility of STRRs imaging detecting active disease in pulmonary fibrosis and opening the possibility to include this technique not only for controlling the response to the therapy, but selecting those who might benefit from somatostatin analog treatment or combined approaches in which ^68^Ga-DOTANOC may function as a carrier for specific antifibrotic drugs or cytotoxic isotopes.

The evidence that ^111^In-DTPA octreotide is highly concentrated in the orbits of patients with Graves’ ophthalmopathy indicated new frontiers in the diagnostic work-up of the disease [46]. Somatostatin receptors, especially SSTR-2 and SSTR-5 subtypes, with high affinity for octreotide [66] are found in retro-ocular muscles and retrobulbar fat of Graves’ ophthalmopathy. During the active phase of disease, activated lymphocytes also express somatostatin receptors. Several studies [39, 41] have already shown that scintigraphy with indium-111 pentetreotide can reveal massive orbital uptake of this analogue of somatostatin in cases of thyroid-associated ophthalmopathy, in contrast to other non-thyroid causes of exophthalmia. Because of the kinetics of the radiotracer and because of the increased blood flow occurring in active thyroid-associated ophthalmopathy, the early pentetreotide accumulation is high and, according to Kahaly et al. [41], could be a sensitive criterion of active disease. Thyroid-associated ophthalmopathy (TAO) is one of the most difficult autoimmune disorders to investigate. The clinical definition of the activity of the ophthalmopathy at the initial presentation is crucial to the identification of the correct therapeutic approach [67]. The treatment of TAO is based on immunosuppressive therapy, such as the use of corticosteroids or radiation, or surgical therapy [68]. Immunosuppression is considered beneficial only in the early active stage, whereas surgery represents the treatment for the end stage of the disease. On the basis of this evidence, a new approach, using labeled somatostatin analogues intended to highlight the presence of activated T lymphocytes in orbital tissue, was attempted in order to identify the early stage of TAO, which should be particularly sensitive to immunosuppressive treatment [69]. Although the evidence to date is characterized by series with lack of standardized methods for evaluating the scintigraphic findings, there is a common trend to demonstrate a potentially relevant role for SRS in the pretreatment evaluation of TAO [70], showing for instance a positive correlation between clinical activity score and ^111^In-octreotide uptake in patients with TAO [40, 46]. Similarly, others [39] reported that visual semi-quantitative analysis of 4 h/24 h planar images during SRS was correlated with the ophthalmologic progression. As the presence of activated lymphocytes in the orbit represents the first requisite for the effectiveness of corticosteroid therapy and as it can be revealed by SRS, recently, this approach was attempted in patients with TAO. The results of orbital ^111^In-octreotide uptake predicted the response to corticosteroid therapy in these patients [38]. Nocaudie et al. [45] found that all patients with severe thyroid ophthalmopathy with positive ^111^In-DTPA Octreotide results showed clinical improvement at 6 months, whereas patients with negative SRS results had not improved. Although a larger group of patients should be investigated before definitive conclusions can be drawn, SRS seems to be a useful method for predicting the clinical response to immunosuppressive treatment in patients with TAO. This might avoid the use of corticosteroid therapy in patients for whom it would have only adverse effects, and might delay surgical treatment for those patients who could benefit from other kinds of medical therapy. Furthermore, some authors [41, 44] demonstrated how ^111^In-octreotide uptake in both orbits was significantly lower after corticosteroid and irradiation treatment [41] and after immunosuppressive therapy [43].

The experience reported in sarcoidosis has the biggest sample of patients (275). In cardiac sarcoidosis, SSTRs might allow for direct assessment of disease activity, especially in the course of treatment. While the current gold standard MRI depicts structural changes like cardiac damage and scarring and edema with the highest spatial resolution, ^68^Ga-DOTATOC uptake may directly reflect the underlying immunological cell activity. In the future, given the complementary nature of PET and MRI signals, the combination of the two may be the optimal diagnostic approach, preferably by integrated MRI/PET. Additionally, whereas a recent study [56] comparing ^68^Ga-DOTANOC and ^18^F-FDG PET/CT has demonstrated encouraging diagnostic accuracy for SSTR-directed PET, the prognostic value of ^68^Ga-DOTATOC PET/CT, especially in comparison to ^18^F-FDG has to be clarified in future trials. Sarcoidosis is a multisystem granulomatous disorder, most frequently involving the lungs, skin, or eyes. Somatostatin receptor scintigraphy (SRS) can visualize sarcoid granulomas through binding of a radionuclide-coupled somatostatin analog to somatostatin receptors that are expressed in sarcoidosis. Somatostatin receptor subtype 2 (SST2) is highly expressed in sarcoid granulomas and used as a substrate for somatostatin receptor scintigraphy (SRS) with ^111^ In-DTPA octreotide and the pendant in PET imaging with ^68^Gallium-labeled somatostatin analogs [71, 72]. The use of SSTR-targeted radiotracers to assess sarcoidosis disease activity is not a novel idea. Giant cells, epithelioid cells, and lymphocytes constitute the bulk of active inflammatory cells in sarcoid granulomas and express SSTRs abundantly [56]. ^68^Ga-DOTANOC PET/CT detected significantly more sites than ^67^Gallium scintigraphy (*P* 0.00 l), especially for thoracic and central nervous system involvement, and appeared more accurate for evaluation of disease activity. ^68^GaDOTATOC-PET was also used and was superior to ^67^Ga in visualizing lesions in the uvea and muscle as well in the lymph nodes in patients with proven sarcoidosis. Three reports [52–54] demonstrated the utility of ^111^In-pentreotide detecting extrapulmonary disease involvement in sarcoidosis and its superiority related to ^67^Gallium nevertheless neither the degree of radioactive accumulation nor a specific pattern of pathological uptake was correlated with disease severity or clinical course, and the SSTRs augmented the yield for thoracic localization in 30% and 14% of the patients for X-rays and CT respectively. There is one report [57] that used 99mTcHYNIC-TOC in sarcoidosis patients and compared the results with laboratory tests, and although the SSTRs clearly could discriminate between positive and negative studies, further studies are needed to find the utility of those results. The evidence about the utility of radiolabeled somatostatin receptor in granulomatous diseases is weak, and although in references found the authors have reported increase uptake of the tracer in the inflammatory focus and visualization of granuloma sites with ^111^In-pentetreotide, the use of radiolabeled somatostatin receptor in patients with granulomatous disease, such as sarcoidosis, tuberculosis, and Wegener’s granulomatosis [73] has also been previously reported; further studies would be important to validate the technique tested with good reference tests and with new radiolabeled somatostatin receptors as with ^68^Ga tracers. The characteristics of ^67^Gallium, as far as lower lesion contrast, the physiological uptake, and the photon energy, are overcome by the characteristics of SSTRs, making it a promising alternative to scintigraphy for evaluating the extend of sarcoidosis [53].

The invasive nature of endomyocardial biopsy has led to a search for alternative diagnostic modalities for the detection of cardiac allograft rejection. The rejection process usually presents with lymphocyte infiltration with or without myocyte necrosis, which indicates the severity of cardiac allograft rejection and the necessity of treatment. Activated lymphocytes express somatostatin receptors; thus somatostatin receptor imaging could be used to target them. The published experience shows the attractive possibility to screen cardiac graft rejection. It is a series of cases with the limitation of the radioligand labeled with ^111−^In, with a source of bias, misclassification, explained by the technical limitations of the index test, not only because the lack of a validated method to evaluate the findings but the image characteristics of the ^111^In as radioligand. It would be interesting to conduct a prospective study by using ^68^Ga compounds.

The upper and lower respiratory tract are common targets in antineutrophil cytoplasmic antibody (ANCA)-associated small vessel diseases (AASV) such as Wegener’s granulomatosis (WG), microscopic polyangiitis (MPA), and the Churg–Strauss syndrome (CSS). Although ANCAs have been proven as an important diagnostic tool [74], it has to be emphasized that, especially in limited AASV, a negative ANCA result does not exclude the diagnosis of active WG or MPA [75]. In AASV, activated T cells are believed to play a central role in pathogenesis [76] and the prominence of T cells and monocyte–macrophages has been demonstrated in lung [77] ear, nose, and throat (ENT) [78] and kidney specimens of active AASV [79]. The promissory results of Neuman’s experience highlight the potential value of SSTR scintigraphy as a non-invasive diagnostic procedure that could detect active disease early in the course of (AASV) and register disease extent, reflecting also response to treatment.

## Conclusions

The evidence summarized in this systematic review highlights the promising results of the potential value of SSTR scintigraphy to detect active disease in different inflammatory conditions mediated by activated somatostatin receptors; although the lack of standardized methods for evaluating the parameters in the index test was the most common characteristic in the majority of the series, there was a clear trend to report positive results related to activated somatostatin receptors in the different organs affected by the inflammatory conditions. In this review, the most solid results had to do with the potential in the atherosclerotic plaque field where undoubtedly the contribution of nuclear medicine by PET/CT systems provided important information about the prediction of acute cardiac events. Because of the methodological limitations in the other series, the results must be rigorously analyzed without ignoring the probity of the molecule.

As a future perspective, it is meaningful to encourage the scientific community to advocate the conduction of large and robust prospective studies with the intention of validating the technique in different scenarios; henceforth, considering the inclusion of this tool in different decision-making trees for diagnostic and prognostic purposes.
